# A Method for the Detection of Tire Wear Microplastics in Zebrafish Guts by Laterally Resolved LA-ICP-MS-Based Elemental Fingerprinting and Chemometrics

**DOI:** 10.1021/acs.analchem.5c07035

**Published:** 2026-04-15

**Authors:** Lukas Brunnbauer, Šimon Juračka, Michaela Vykypělová, Lucie Vrlíková, Elisabeth Eitenberger, Pavel Pořízka, Ondrej Adamovsky, Jozef Kaiser, Gabriela Kalčíková, Andreas Limbeck

**Affiliations:** † Institute of Chemical Technologies and Analytics, 425555 TU Wien, Getreidemarkt 9/164-I2AC, Vienna 1060, Austria; ‡ Faculty of Mechanical Engineering (FME), Brno University of Technology, Technická 2896/2, 616 69 Brno, Czech Republic; § RECETOX, Faculty of Science, Masaryk University, Kotlářská 2, Brno 611 37, Czech Republic; ∥ Institute of Animal Physiology and Genetics, Czech Academy of Sciences, Veveří 97, 602 00 Brno, Czech Republic; ⊥ Central European Institute of Technology (CEITEC), Brno University of Technology, Purkynova 656/123, 612 00 Brno, Czech Republic; # Faculty of Chemistry and Chemical Technology, 112794 University of Ljubljana, Večna pot 113, Ljubljana 1000, Slovenia

## Abstract

Tire wear particles (TWPs) are generated by mechanical abrasion of tires on road surfaces and represent a significant source of microplastic pollution, contributing an estimated 30–50% of total microplastic emissions in Europe. Due to their persistence and limited biodegradability, TWPs accumulate in terrestrial, aquatic, and atmospheric environments. However, their detection and quantification remain challenging: carbon black hampers FTIR analysis, while pyrolysis-GC-MS yields only bulk mass data without information about particle abundance or size distribution. We present a novel approach to address this gap using laser ablation-inductively coupled plasma-mass spectrometry (LA-ICP-MS) combined with elemental fingerprinting and machine learning. We apply this method to zebrafish gut tissue to differentiate TWPs from biological tissue, paraffin-embedded material, and other naturally occurring particles. A random forest model trained on multielement signatures enables pixelwise classification of imaging data recorded with 7-μm lateral resolution despite the complexity of both TWP and biological matrices. Our results demonstrate the potential of LA-ICP-MS elemental imaging as a sensitive tool for TWP detection in biological tissue, providing new opportunities for monitoring and ecotoxicological studies.

## Graphical abstract

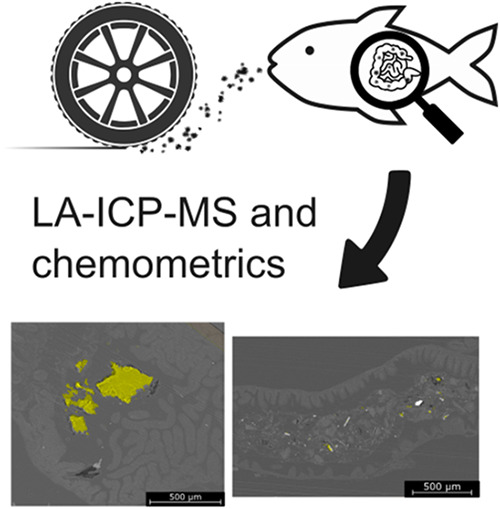

## Introduction

Tire wear particles (TWPs) are increasingly recognized as a major yet often overlooked source of microplastic pollution in terrestrial, atmospheric, and aquatic systems.^1,2^ Estimations vary, but TWPs likely account for a substantial fraction of the environmental microplastics. In fact, the contribution of TWPs to total microplastic emissions in European countries has been reported to range from about 30% to over 50%.^2,3^

TWPs are generated through the mechanical abrasion of tire treads on road surfaces.^4^ This wear process releases a complex mix of synthetic rubbers, fillers, and road pavement fragments. Generation of TWPs depends on several factors, including vehicle type, tire composition, road surface characteristics, and driving behavior (e.g., acceleration, braking, and cornering).^1,5–7^ Notably, heavier vehicles contribute disproportionately more TWPs, and thus the support of heavier electric vehicles under the European Green Deal, despite their benefits for air emissions, may inadvertently increase TWPs. The emission of TWPs into the environment is assessed to be approximately 10% of the tire mass during use.^8^ Per capita emissions are estimated to range from 0.23 to 4.7 kg per year, with a global average of 0.81 kg per year.^5^

Once released into the environment, TWPs are not well biodegradable,^9^ and thus persist in soil, water, air, and sediments.^2^ However, monitoring their environmental levels is considerably difficult. Conventional microplastic monitoring techniques, such as Fourier transform infrared (FTIR) microscopy, have difficulties in detecting TWPs due to the carbon black content, which absorbs infrared radiation and prevents transmission. Therefore, pyrolysis-gas chromatography–mass spectrometry (py-GC-MS) is often used to quantify TWPs in environmental and biotic samples.^10^ However, this method provides only mass-based measurements, leaving unanswered the questions of particle abundance and size distribution, parameters that are essential for assessing their ecotoxicity and potential effects on human health. As a result, general monitoring campaigns often underestimate the presence of TWPs. This methodological gap underscores the need for novel techniques.

This work aims to apply laser ablation-inductively coupled plasma-mass spectrometry (LA-ICP-MS)-based imaging to identify TWPs in a biological matrix, namely, zebrafish guts, using elemental fingerprinting. Elemental fingerprinting is commonly used for classification tasks ranging from origin determination of food products^11–14^ to forensic applications^15,16^ using ICP-based techniques. The advantage of ICP-MS for these tasks is its outstanding sensitivity and a linear working range covering several orders of magnitude, enabling detection of major, minor, and trace constituents as well as multielement detection capabilities. This allows classification based not only on a single marker element but also on the development of more complex machine learning models to distinguish between samples based on the signals of multiple elements. When analyzing polymers with ICP-MS, only the distinct two-phase sample transport of carbon can be used to distinguish between polymer types.^17^ Nevertheless, for elemental fingerprinting, the selection of elements is often based on inorganic contaminations or additives^18^ present in different polymer types.^15,19^ Combining ICP-MS with Laser Ablation (LA) enables laterally resolved analysis with low-μm resolution. Applying the concept of elemental fingerprinting allows us to classify each pixel of the obtained image.

In this work, we develop a method to distinguish between TWPs and the tissue of zebrafish guts and potential naturally occurring particles in the guts based on their unique elemental pattern. This is challenging due to the complex elemental fingerprint in both biological tissue and environmental TWPs since these are not well-defined matrices, and a significant variance is expected. Therefore, in a first step, we apply unsupervised clustering methods to evaluate the feasibility of distinguishing TWPs from zebrafish tissue and a range of potential occurring particles in the zebrafish gut based on a selection of detected elements. Next, we train and optimize a random forest (RF)-based classification model and evaluate its performance using independent test data. Finally, we applied the model to identify and detect TWPs in zebrafish guts.

## Experimental Section

### Preparation of Tire Wear Microplastics

The TWPs were obtained from a local auto repair shop by mechanically abrading the tread layer of old, used tires, mimicking the processes that occur during tire use. The generated material was sieved through a 500-μm stainless steel mesh to remove coarser particles. The characteristics of the resulting TWPs have been described in detail in our previous studies.^9,20^ The particle shape and surface morphology were found to be irregular with a complex surface texture with a surface area of 34 cm^2^ g^–1^. In this study, the particle size range was further narrowed by sieving the TWPs with 80 and 355 μm meshes, and the fraction between these sizes was used for the experiments. Particle size distribution was measured using a Microtrac S3500 Bluewave laser diffraction analyzer, yielding a particle size of 80 ± 39 μm. The size distribution of the particles can be found in the Supporting Information (Figure S1). It should be noted that the selected particle size range reflects typical sizes reported in toxicological studies of TWPs and zebrafish.^21–23^

### Exposure of Zebrafish

Two adult zebrafish were euthanized and subsequently gavaged with TWPs (1 mg in 100 μL). Using optical microscopy, we determined that 1 mg of TWPs corresponds to 5109 ± 644 particles (n = 10). This number is in the range of particles found accumulated in zebrafish guts in toxicological studies.^22,24^ Prior to administration, the particles were hydrated in zebrafish husbandry water and shaken at 130 rpm for 24 h to ease the manipulation.

In brief, the zebrafish were euthanized using Tricaine Methanesulfonate (MS-222, Merck; 0.3 mg/mL). The particles were delivered into the gastrointestinal tract using ultrafine pipette tips. The tips were slightly cut to enlarge the hole to ensure the smooth passage of the TWPs. The pipette tip was carefully inserted into the esophagus to avoid tissue perforation. Fish were then dissected, and the entire intestine was dissected and placed in histological cassettes. Samples were fixed in 4% formaldehyde in Phosphate buffered saline (PBS) and stored at 4 °C until further processing.

All procedures were approved by the Ethical Committee of the Czech Ministry of Education, Youth and Sports (approval number: MSMT-12088/2024-4).

### Paraffin Embedding of Zebrafish

Samples of zebrafish digestive tract were fixed in tissue cassettes for a minimum of 24 h in 4% paraformaldehyde at 4 °C, following standard histological protocols. Fixation was carried out to prevent tissue autolysis and to ensure optimal preservation of the cellular and structural morphology. After fixation, the tissues were washed overnight in running water to remove residual fixative.

The cassettes were subsequently transferred with forceps into glass containers containing a graded ethanol series, followed by xylene, in order to achieve progressive dehydration and clearing of the tissues. The tissues were incubated in each ethanol bath for defined periods of time according to the protocol (30% ethanol for 3 h, 50% ethanol for 3 h, 70% ethanol overnight, 80% ethanol for 1 h, 95% ethanol for 1 h, and 100% ethanol for 1 h). Following dehydration, the tissues were immersed in three successive xylene baths. As a result of the clearing process, the tissues became partially transparent, which facilitated effective paraffin infiltration.

After dehydration and clearing, the samples were immersed in molten paraffin wax in a thermostat maintained at 56 °C. Each sample was carefully oriented in a prewarmed metal mold and embedded in liquid paraffin. A labeled embedding ring was then positioned on the mold to ensure proper identification. The paraffin blocks were allowed to solidify at room temperature for approximately three h prior to further processing.

In addition to biological samples, the TWPs themselves were also embedded in paraffin. These particles were not subjected to fixation, dehydration, or clearing; instead, they were directly mixed with molten paraffin and cast into molds, resulting in paraffin blocks that served as control samples.

To assess the feasibility of identifying TWPs in zebrafish guts, in the first step, bulk elemental fingerprints of TWPs and potential other (naturally) occurring particles in zebrafish guts are compared. Therefore, prepared TWPs, Hikari feed (Hikari Tropical Micro Pellets) and dried artemia used to feed the zebrafish as well as certified reference materials obtained from the European Commission, Joint Research Centre (JRC), Geel, Belgium: road dust (BCR-723R), mussel’s tissue (BCR-278R), lake sediment (BCR-280R), river sediment (BCR-684), were evenly mounted in paraffin for representative bulk analysis of the elemental fingerprint. Other potential microplastics (PVC, PET, PP, HDPE) sourced from consumer plastics were milled, sieved (80–335 μm) and mounted in paraffin as well. Furthermore, a blank gut and blank paraffin were analyzed, resulting in a total of 13 materials for the preliminary experiments.

### Microtome Cutting

Obtained paraffin blocks were cut using a commercial rotary microtome (M-240, Myr, Tarragona, Spain). Therefore, each block was cut in increments of 10 μm with frequent visual inspection using an optical microscope (VHX-5000, Keyence) to assess if a proper cross section of the gut for analysis had been obtained. In contrast to conventional sample preparation of paraffin-embedded samples using a microtome, we did not analyze the individual sections, but rather the cross-section of the block itself.

### Scanning Electron Microscopy

After visual inspection of freshly cut paraffin blocks using an optical microscope (VHX-5000, Keyence, Osaka, Japan), 5 nm of gold was sputtered on the sample surface using a sputter coater (Agar Scientific, Rotherham, UK) for SEM analysis. Additionally, gold was used as a marker element in the LA-ICP-MS analysis to identify the start and end of each line scan in the transient data, facilitating synchronization with the laser log file and the following data evaluation. Surface sensitive analysis of the morphology of the samples to confirm the presence of particles in the cross sections was carried out using a scanning electron microscope (SEM, FEI Quanta 2050 FEG) in low vacuum mode and an acceleration voltage of 20 kV in backscattered electron mode (BSE).

### LA-ICP-MS Analysis

LA-ICP-MS measurements were performed using an imageGEO193 laser ablation system (ESL, Bozeman, Montana, US) operating at a wavelength of 193 nm and equipped with a TwoVol3 ablation chamber. The laser ablation unit was connected to an iCAP Qc ICP-MS (ThermoFisher Scientific, Germany) via Tygon tubing (inner diameter: 1.6 mm). Ablation was conducted under a continuous helium flow of 0.8 L min^–1^. Argon, supplied at 1 L min^–1^ as a makeup gas, was combined with the sample aerosol immediately before entering the ICP using a dual concentric injector (DCI) (ESL, USA). This setup and experimental conditions result in a washout time of <100 ms. The instrument was tuned daily, maximizing ^115^In signal while keeping the oxide ratio ^232^Th^16^O/^232^Th < 1% while ablating NIST612 (Standard Reference Material, National Institute of Standards and Technology, Gaithersburg, MD).

To get an overview of elements present in TWPs that could act as marker elements, the *m*/*z* range of 6–238 was scanned using a dwell time of 10 ms per *m*/*z*. Therefore, a plateau-like signal was introduced to the ICP-MS using a line scan with a 70-μm spot size, a laser fluence of 2.4 J/cm^2^, a scan speed of 700 μm/s, and a repetition rate of 50 Hz. The specified *m*/*z* range was scanned 100 times and averaged to obtain representative signals.

For bulk analysis of potential naturally occurring particles and other microplastics in zebrafish guts, 20 parallel line scans (length of 5 mm) were ablated for each of the investigated materials (road dust, mussel’s tissue, lake sediment, river sediment, paraffin, gut tissue, hatched Artemia, Hikari feed, PVC, PET, PP, HDPE, and TWPs). Using a spot size of 70 μm, a laser fluence of 2.4 J/cm^2^, a scan speed of 700 μm/s, and a repetition rate of 50 Hz results in an ablation time of around 7 s per line. These settings resulted in a continuous representative signal for each sample. For each line scan, the ICP-MS signal was averaged and used for data evaluation.

To obtain elemental maps without any artifacts using a spot size of 7 μm, the dwell times of the detected *m*/*z* are selected in a way to obtain a final cycle time of 100 ms of the quadrupole as described by van Elteren et al.^25^ The scan speed of the stage is set to move 7 μm in 100 ms, resulting in 70 μm/s. With this setup, any pixel bleeding should be avoided. To compensate for changes in sensitivity between samples, the obtained signals were normalized to signals obtained from NIST612, which was analyzed before and after each image. It should be noted that measurement times for each image varied between 1 and 3 h, depending on the covered area, and variations between obtained NIST612 signals between measurements were <20%. ICP-MS data were recorded using Qtegra 2.10. The ablation depth after imaging experiments was evaluated using a Dektak XT stylus profilometer (Bruker Corporation, MA) to be 8 μm. An overview of the measurement parameters applied for imaging experiments can be found in Table 1.

**Table 1:** LA-ICP-MS Measurement Parameters for Imaging Experiments

LA		ICP-MS	
laser fluence (J/cm^2^)	2.4	cycle time (ms)	100
spot size (μm)	7	cool gas flow (L/min)	14
scan speed (μm/s)	70	aux. gas flow (L/min)	0.8
repetition rate (Hz)	100	monitored isotopes with dwell times	^13^C (10 ms), ^64^Zn (10 ms), ^48^Ti (10 ms), ^92^ Mo (14 ms), ^121^Sb (14 ms), ^142^Nd (14 ms), ^197^Au (5 ms), ^208^Pb (15 ms)

In this work, 6 samples were imaged: 2 samples of TWP in paraffin, 2 samples of blank guts for model building and evaluation, and 2 guts exposed to TWP. Carbon was monitored as a marker element to facilitate alignment of the laser log file with the transient ICP-MS data in the following data evaluation.

### Data Evaluation

The first step of evaluating LA-ICP-MS data includes background correction, normalizing to NIST612 signals to enable comparison of measurements carried out on different days. Unsupervised analysis of bulk data was carried out using a custom Jupyter Notebook with the sklearn library. Elemental maps from imaging experiments were constructed using Iolite version 4.10.7 by aligning the laser log file with the transient ICP-MS signal. Further, pixel matrices of the obtained elemental maps were imported into ImageLab 4.33 (Retz, Austria). Image registration of elemental maps with SEM pictures was carried out manually using 4–8 reference points per sample. The selection of training and test data for the random forest (RF)-based classifier and the optimization of its hyperparameters are described in more detail in the Results section.

## Results and Discussion

Building on the need for robust analytical tools to identify TWPs in complex biological matrices, we applied a combined LA-ICP-MS and machine-learning workflow to zebrafish gut samples to assess their capability for accurate detection and classification. In developing this workflow, particular attention was given to sample preparation, as conventional tissue digestion approaches often result in microplastic loss or degradation and eliminate crucial spatial information.^26,27^ Also, the particle sizes and number concentrations of TWPs used in this study were selected to reflect environmentally relevant conditions. The size distribution of the applied TWPs (80 ± 39 μm) falls within the range reported in previous toxicological studies investigating TWP exposure in zebrafish.^21–23^ Furthermore, the administered particle numbers (5109 ± 644) were comparable to those reportedly found in zebrafish guts (1037 ± 112) following exposure to environmentally relevant concentrations of 20 mg/L.^22^ The exposure concentration of 20 mg/L falls within the upper range of predicted environmental concentrations (PECs) reported for TWPs in surface waters (0.03–56 mg/L). However, it exceeds most measured concentrations in river systems, which are typically reported in the lower mg/L range (e.g., ∼0.5–6 mg/L).^24^ Thus, 20 mg/L should be interpreted as representing a high-end or worst-case environmental exposure scenario. At these sizes and number concentrations, TWPs caused oxidative stress, transcriptomic deregulation, changing of the gut microbiota, and inhibited growth and development in adult zebrafish.^22^ We also verified that sample preparation (especially exposure to xylene, which is known to penetrate TWPs^28^) does not alter the elemental composition of TWPs (see Figure S2).

### Elemental Fingerprint of TWPs

In the first step, the elemental fingerprint, which consists of marker elements for the TWPs, was investigated. Therefore, TWPs mounted in paraffin were continuously analyzed with a large spot size (70 μm) to sample the particles as representatively as possible. Recording the mass spectrum from *m*/*z* 6–238 allowed us to identify potential marker elements in the TWPs. The mass spectrum was compared to the mass spectra of pure paraffin and a gas blank to identify potential marker elements. A wide range of environmental elements, such as C, Na, Mg, K, Ca, Cu, Fe, and Zn, were detected in the TWPs. Additionally, elements of potential anthropogenic origin, such as Ti, Mo, Sb, Nd, and Pb, were detected. Isotopic patterns of each element were verified against their natural abundances to ensure accurate identification and avoid interference-related misinterpretation.

Most biogenic elements are expected to be abundant in zebrafish tissue, as well, and thus unsuitable for distinguishing TWPs from tissue. Consequently, Zn, Ti, Mo, Sb, Nd, and Pb were selected for elemental fingerprinting. Even though Zn is a biogenic element, it was selected because it is one of the main additives (as ZnO and Zn stearate) in tires and is typically present at the g/kg level. Pb is known to occur as a contaminant in ZnO and can, therefore, be found in TWPs as a trace constituent (mg/kg). Ti serves as a catalyst in rubber manufacturing and can also be present as an additive.^29^ Sb and Mo are commonly found in commercial brake pads and have been detected in several studies in road dust and TWPs.^30–32^ Nd-based Ziegler–Natta catalysts are employed in the synthesis of diene-based elastomers for tire applications.^33^

To assess the feasibility of elemental fingerprinting to identify TWPs in zebrafish guts and reliably distinguish them from naturally occurring particles, in a first step, bulk analysis is carried out. Therefore, the elemental fingerprint of TWPs was compared to 12 other compounds (lake sediment, river sediment, road dust, mussel tissue, Hikari feed, and dried artemia used to feed the fish) as well as a blank zebrafish gut and paraffin. Additionally, other microplastics (PVC, PET, PP, and HDPE) were analyzed. For each sample, 20 line-scan measurements were carried out, ICP-MS signals were averaged, and data were standardized for the following unsupervised data evaluation. In a first step, principal component analysis (PCA) was calculated, and biplots for PC1 vs PC2 (Figure 1a) and PC2 vs PC3 (Figure 1b) are shown. The detected elements were the input variables. PCA enables visualization of the 6-dimensional feature space via projections and enables interpretability based on the loadings. Clear differences between the analyzed samples can be observed based on the selected elements. PET stands out with a high signal for Sb due to a common Sb-based catalyst used in PET manufacturing.^34^ Lake sediment stands out with high signals for Ti, whereas river sediment shows high signals for Nd. Road dust shows significant signals for Pb and Mo, and as expected from the additives, TWP shows high amounts of Zn. In the biplots, TWPs are well separated from the other compounds. To provide further insights into the elemental patterns of the different materials, a standardized (column-wise) heatmap of the observed signals is provided in the Supporting Information (Figure S3).

**Figure 1:**
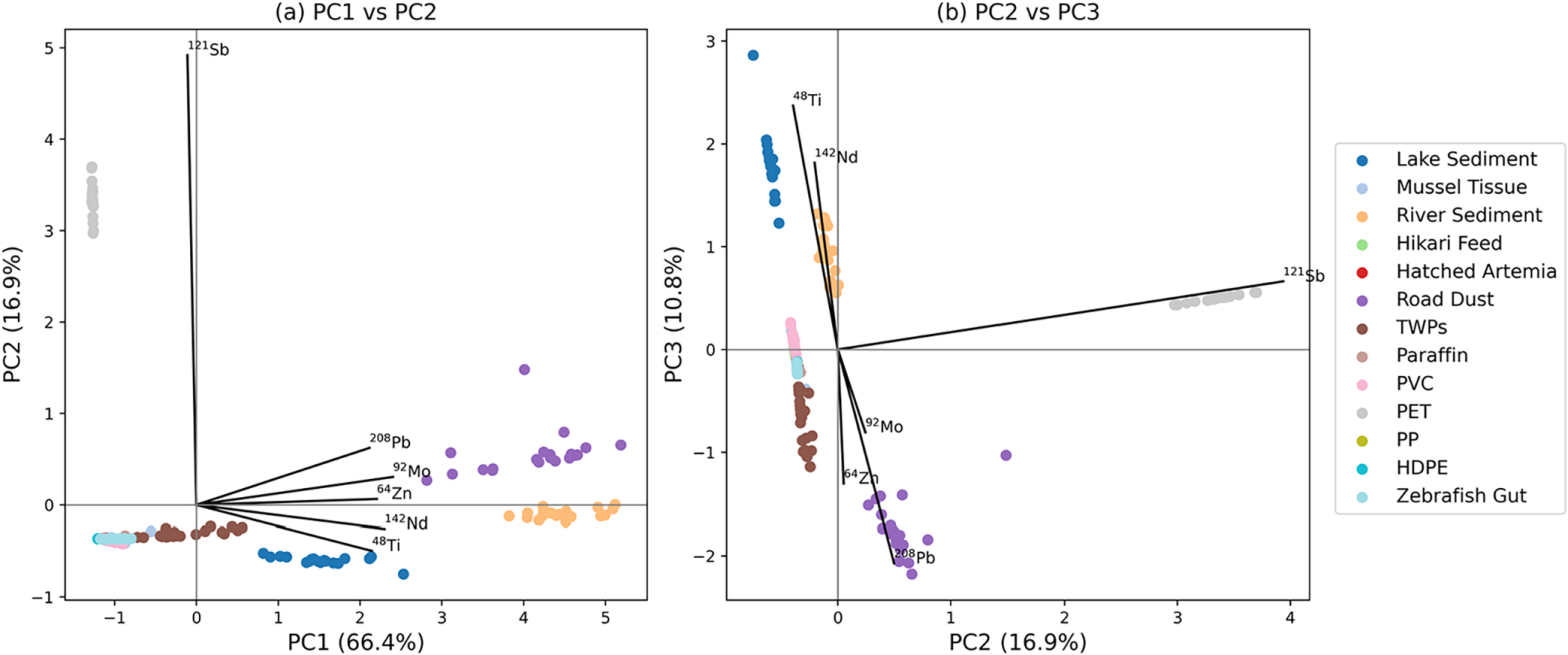
PCA for the bulk analysis of potential (naturally) occurring particles/organics in zebrafish guts and TWPs (a) shows the biplot for PC1 vs PC2, (b) shows the biplot for PC2 vs PC3.

For further investigations, hierarchical cluster analysis (HCA) using Ward’s method and the Euclidean distance is applied to the data set, with the detected elements being the input variables (Figure 2). Compared with PCA, HCA represents the full feature space and does not rely on projections. Nevertheless, the interpretability of which elements contribute to differentiation between samples is limited. The obtained dendrogram shows two distinct clusters, with cluster 1 covering samples with a high content of inorganic components: lake sediment (1a), river sediment (1b), and road dust (1c). Cluster 2 covers the samples containing mostly organic compounds: PET (2a) stands out in this cluster, most likely due to its high Sb content. Mussel tissue, PVC, Paraffin, HDPE, PP, Zebrafish gut, Hikari feed, and hatched Artemia are all combined in cluster 2b. In cluster 2, a clear difference between TWPs (2c) and the other samples is observed, indicating that distinguishing between TWPs and potential naturally occurring particles and microplastics, as well as paraffin used for sample fixation and tissue, is feasible based on the selected elements.

**Figure 2:**
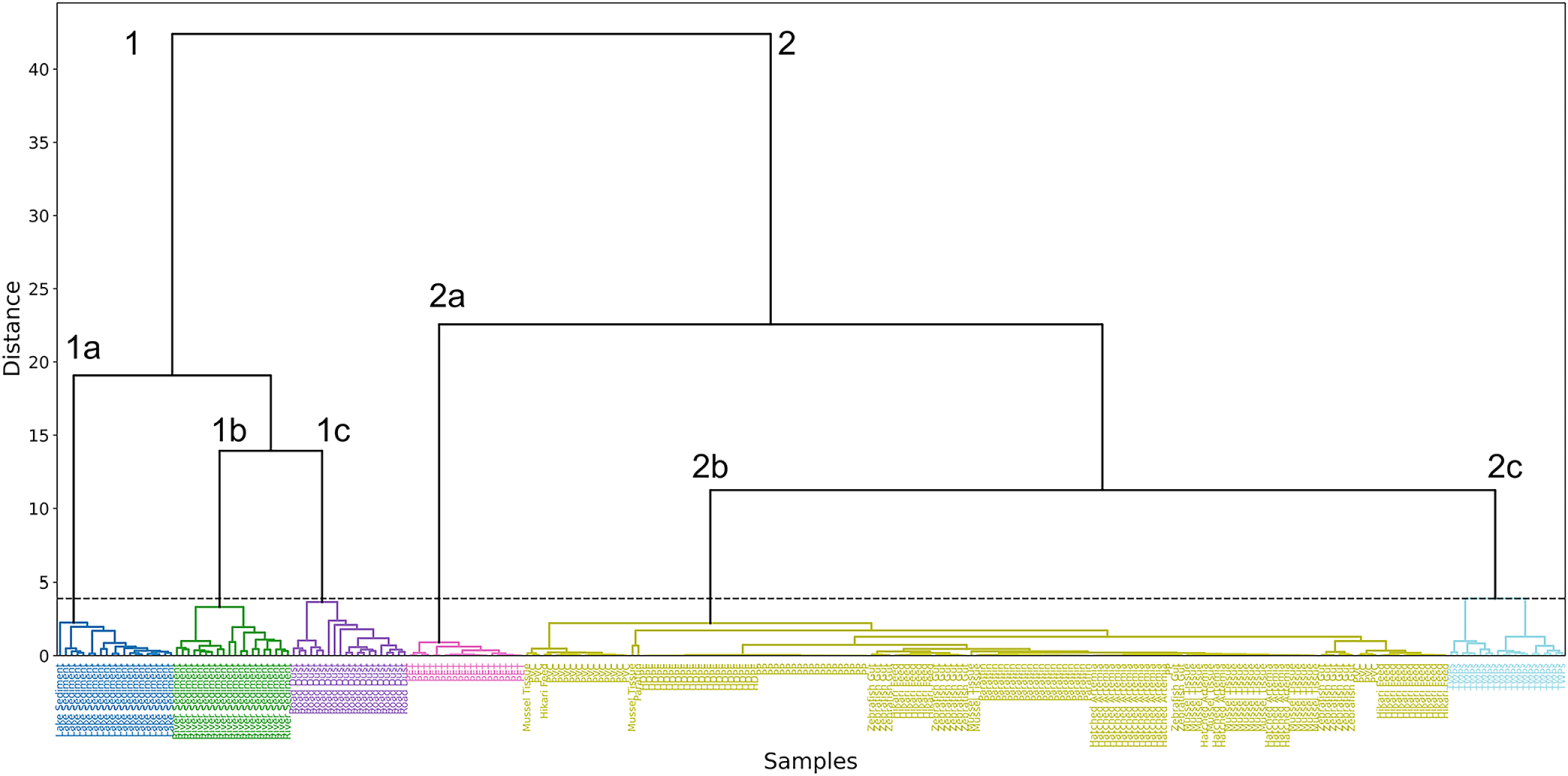
Dendrogram obtained from the HCA using Ward’s method and Euclidean distance. Cluster 1 corresponds to samples with a large fraction of inorganic compounds and subdivides into clusters 1a covering lake sediment, 1b covering river sediment, and 1c covering road dust. Cluster 2 corresponds to samples with a large fraction of organic compounds and subdivides into 2a PET, 2b mussel tissue, PVC, Hikari feed, HDPE, PP, zebrafish gut, paraffin, and hatched artemia. Cluster 2c covers TWP.

Prior to the detection of TWPs in the gut, preliminary analyses were conducted, including the examination of elemental maps for the selected marker elements in TWPs embedded in paraffin and in blank gut samples (Figures S4 and S5). Further, elemental maps were overlaid with SEM images to correlate the signals with the position of the particles in paraffin (Figure 3 left column) and the elemental distribution within a blank gut (Figure 3 right column). For each element, the range of the color scale is selected according to the distribution of pixel values in the TWP sample. A threshold value is defined as threshold_element_ = 0.75 * *P*_(95,element)_ with *P*_95_ being the 95th percentile of the data of the image. Only pixels exceeding this threshold are marked in the corresponding images. This ensures that the obtained signals between the TWP in paraffin and the blank gut can be easily compared visually for each element.

**Figure 3:**
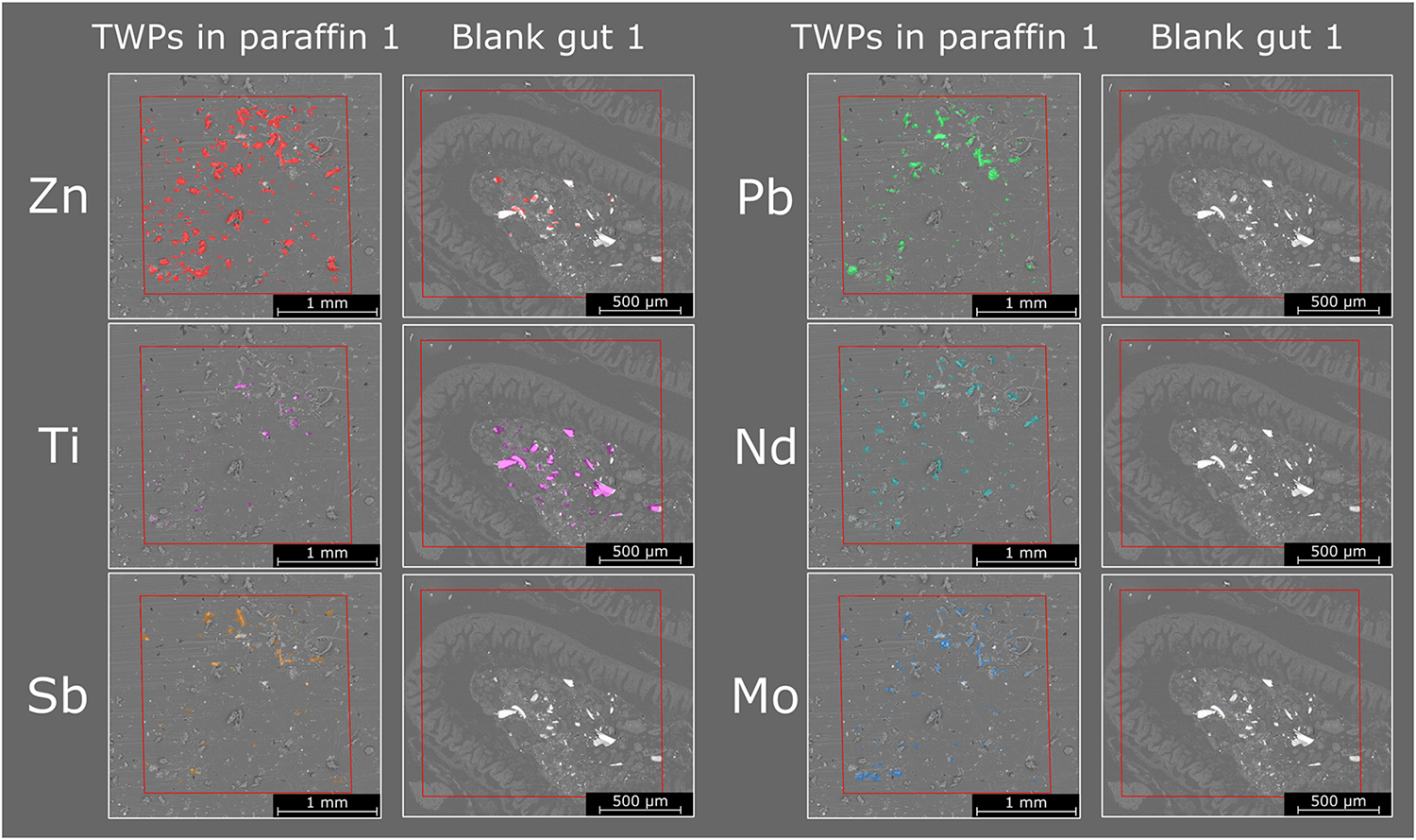
Elemental maps for Zn, Ti, Sb, Pb, Nd, and Mo measured for TWPs in paraffin (left column) and a blank gut (right column). Elemental maps are overlaid with SEM images. The analyzed areas are marked in red.

Zn signals showcased its capabilities as a marker element for TWP due to its high concentration and presence as an additive. Overlaying the Zn distribution with the SEM image enables clear identification of the position of the TWP in the image. Nevertheless, certain parts of the blank gut exhibit similar signal levels of Zn within the sample, which rules out using just Zn as an individual marker element for TWP in the guts. Besides Zn, all other elements are detected only within some individual TWP. Importantly, the TWPs analyzed in this study originated from multiple tires and potentially different manufacturers, making the sample highly environmentally relevant and reflective of real-world conditions,^35^ where diverse additives and incorporated road dust generate distinct elemental fingerprints for individual particles. It should be noted that, similar to Zn, some of the selected elements were found at similar levels in the blank gut as in the TWPs (Ti, Pb), while other elements (Sb, Nd, Mo) were not found at similar levels, but they were not present in all TWPs. This indicated that there is no single marker element capable of clearly distinguishing between the blank gut and the individual TWPs. Therefore, in the next section, all 6 elements (Zn, Ti, Mo, Sb, Nd, and Pb) identified as marker elements for TWPs are considered to build a multivariate classification model. It should be noted that even though Ti was confirmed not to be influenced by major interferences in TWP based on the isotopic pattern, the selected isotope ^48^Ti may be interfered by ^48^Ca in the gut samples.

### Training a Random Forest-Based Classification Model

For training and evaluation of the performance of a random forest (RF)-based classification model, samples of TWPs in paraffin and blank guts were used. Therefore, 2 samples of TWP in paraffin and 2 samples of blank guts were imaged. The samples “TWP in paraffin 1” and “blank gut 1” were used to extract training data and train the classification model. The model’s performance was evaluated by applying it to test data extracted from “TWP in paraffin 2” and “blank gut 2”. Therefore, the performance is evaluated on the basis of independent test data. Elemental maps for those samples can be found in the Supporting Information (Figures S6 and S7).

A binary RF-based classification model was trained by providing training data for TWPs (class 1) and zebrafish gut and paraffin (class 2). Training data for TWPs (class 1) was extracted from images of “TWPs in paraffin 1” by selecting 4000 random pixels with Zn signals above threshold_Zn_ = 0.75 * *P*_(95,Zn)_ (corresponding to 4000 random pixels marked in red in Figure 3). Training data for class 2 (zebrafish gut and paraffin) was obtained by selecting 4000 random pixels of the sample “blank gut 1”. All 6 elements identified as potential marker elements (Zn, Ti, Pb, Nd, Sb, Mo) were input variables for the RF model after standardization. Hyperparameters were optimized by minimizing the out-of-bag (OOB) error as an internal estimate of the generalization error averaged for both classes (number of trees = 65 and *R*-value = 0.58) (Figure 4). The *R*-value specifies the percentage of the training set used to build the individual trees. The procedure was repeated five times with different random seeds, and the results were averaged to ensure stability of the error estimate.

**Figure 4:**
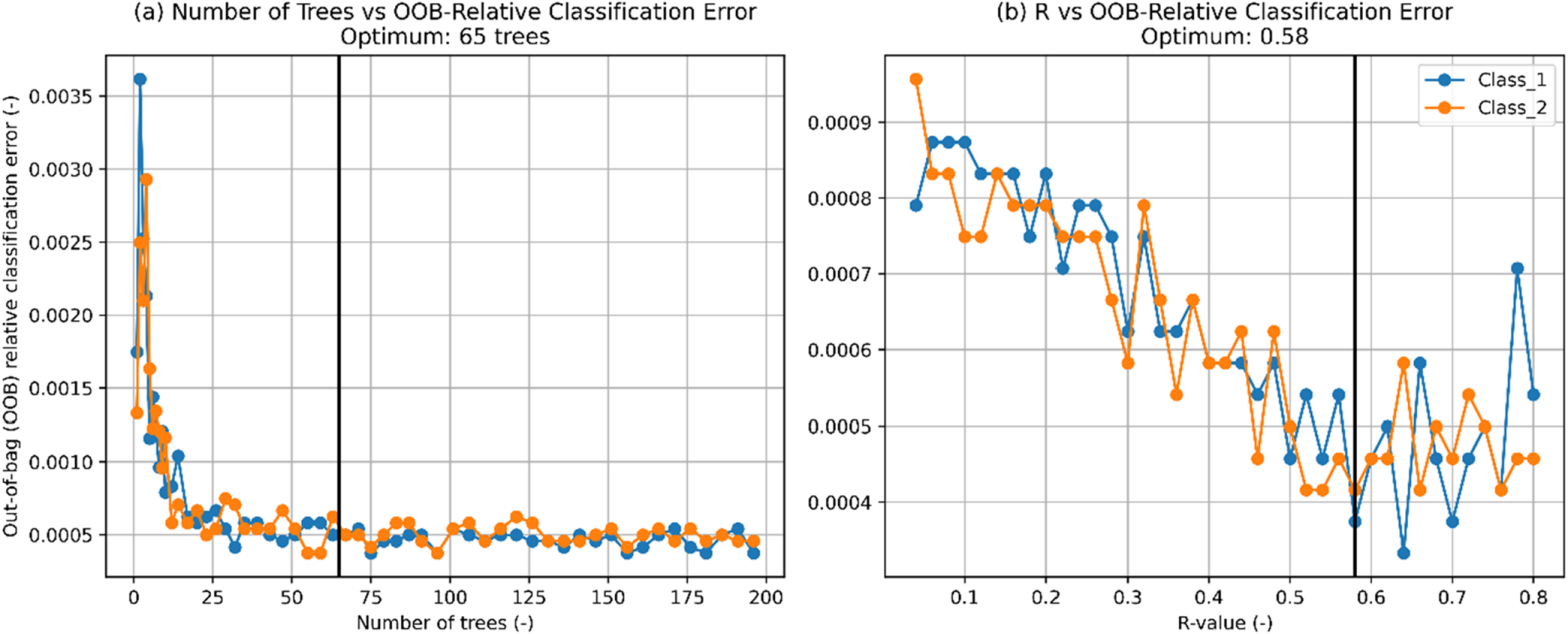
Optimization of the RF hyperparameters: (a) number of trees and (b) *R*-value.

To evaluate the performance of the trained classifier, it is applied to the test data set consisting of “TWPs in paraffin 2” and “blank gut 2”. Similarly, as described above, in the “TWPs in paraffin 2” data sets, 4000 random pixels exceeding the same threshold of Zn were selected as class 1, and 4000 random pixels of “blank gut 2” were selected as class 2. To evaluate the performance, the trained model was applied to the test data set. The results indicate excellent performance of the model with only 9 TWPs pixels (of a total of 4000 pixels) being wrongly classified as tissue and paraffin, while no pixels of tissue and paraffin were wrongly classified as TWPs, showcasing the model’s capabilities to avoid false positives.

Additionally, the classification model was applied to the images of TWPs in paraffin 2 and blank gut 2 and overlaid with the corresponding SEM pictures. The results are shown in Figure 5, with pixels classified as TWPs (class 1) marked in yellow and pixels classified as zebrafish gut and paraffin (class 2) marked as transparent. All particles visible in the SEM picture in the TWP in the paraffin sample were recognized as TWPs by the developed RF model, while no pixels in the blank gut 2 sample were classified as TWPs, demonstrating the model’s capabilities. Importantly, even in the blank zebrafish gut, numerous particles are visible in the SEM images, most likely originating from fish feed or other natural sources. However, the developed RF classification model correctly distinguishes these particles from TWPs.

**Figure 5:**
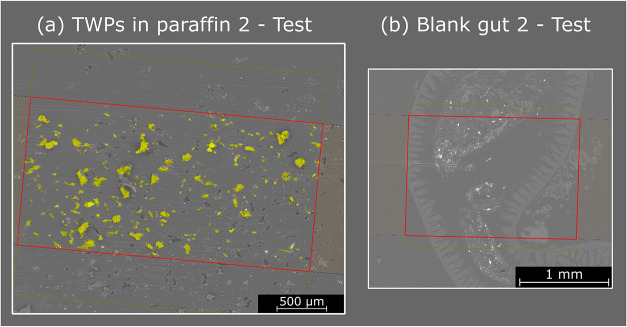
Classification results applied to the test data overlaid with SEM pictures: (a) TWPs in paraffin 2, (b) blank gut 2. Pixels classified as TWPs by the developed RF model are marked in yellow. Pixels classified as tissue and paraffin are transparent. The analyzed area is marked in red.

### Application to Detect TWPs in the Zebrafish Gut

In the previous section, we demonstrated that the model can reliably distinguish between particles and tissue based on LA-ICP-MS elemental images. In the final step, the model was applied to identify TWPs directly in the zebrafish gut. Therefore, we applied the developed model to evaluate elemental maps obtained from zebrafish guts with inserted TWPs (elemental maps for the two analyzed samples are shown in the Supporting Information in Figures S8 and S9). Classification results of the developed model are listed in Figure 6. For the sample TWPs in gut 1, several particles in the center of the sample are identified as TWPs, revealing not only the position but also the shape of the individual particles, which matches the shape of the particles observed in the SEM pictures. In the section of the analyzed gut, no other particles were visible. For the sample TWPs in gut 2, several TWPs were detected using the developed method. Aligning the results with the SEM pictures enabled correlating the classification results with individual particles observed in the SEM pictures. For this sample, similar to the blank samples, many particles were found in the zebrafish gut in the SEM pictures. Combining SEM pictures with LA-ICP-MS-based elemental fingerprinting enabled clear identification of TWPs among the particles found in zebrafish guts. Additionally, it should be noted that the information depth of SEM pictures and LA-ICP-MS analysis is different. While SEM analysis is expected to have an information depth showing only particles in the first 2–4 μm of the sample, LA-ICP-MS analysis ablates 8 μm. Therefore, TWPs that were not fully observed in the SEM picture due to still being (partially) covered by paraffin may be detected by the developed LA-ICP-MS method. Additionally, it should be highlighted that the obtained pixel size for LA-ICP-MS images corresponds to 7 μm. To estimate the smallest detectable particle size, we apply geometric considerations and not a classical LOD approach based on a mass fraction. A classical LOD approach is not feasible in our scenario, since the developed classifier is trained on pure pixels that are fully contained within an individual TWP. Therefore, even if we were able to analytically detect the signal of the marker elements for *a* < 7 μm particle within one pixel, the classifier would not reliably identify the pixel as TWP since the absolute signals would be lower. Therefore, only a particle that produces at least one pixel fully contained within the particle is detectable. Accordingly, based on geometric assumptions and under idealized conditions, the smallest detectable particle size is estimated to be 14 μm, corresponding to twice the laser spot size. This requirement ensures that at least one laser shot is fully contained within the particle, independent of raster alignment. Consequently, 14 μm represents the theoretical lower limit of detectable particle size for the developed method. It should be noted that the practical detection limit may be influenced by additional factors such as particle shape/morphology and beam profile.

**Figure 6:**
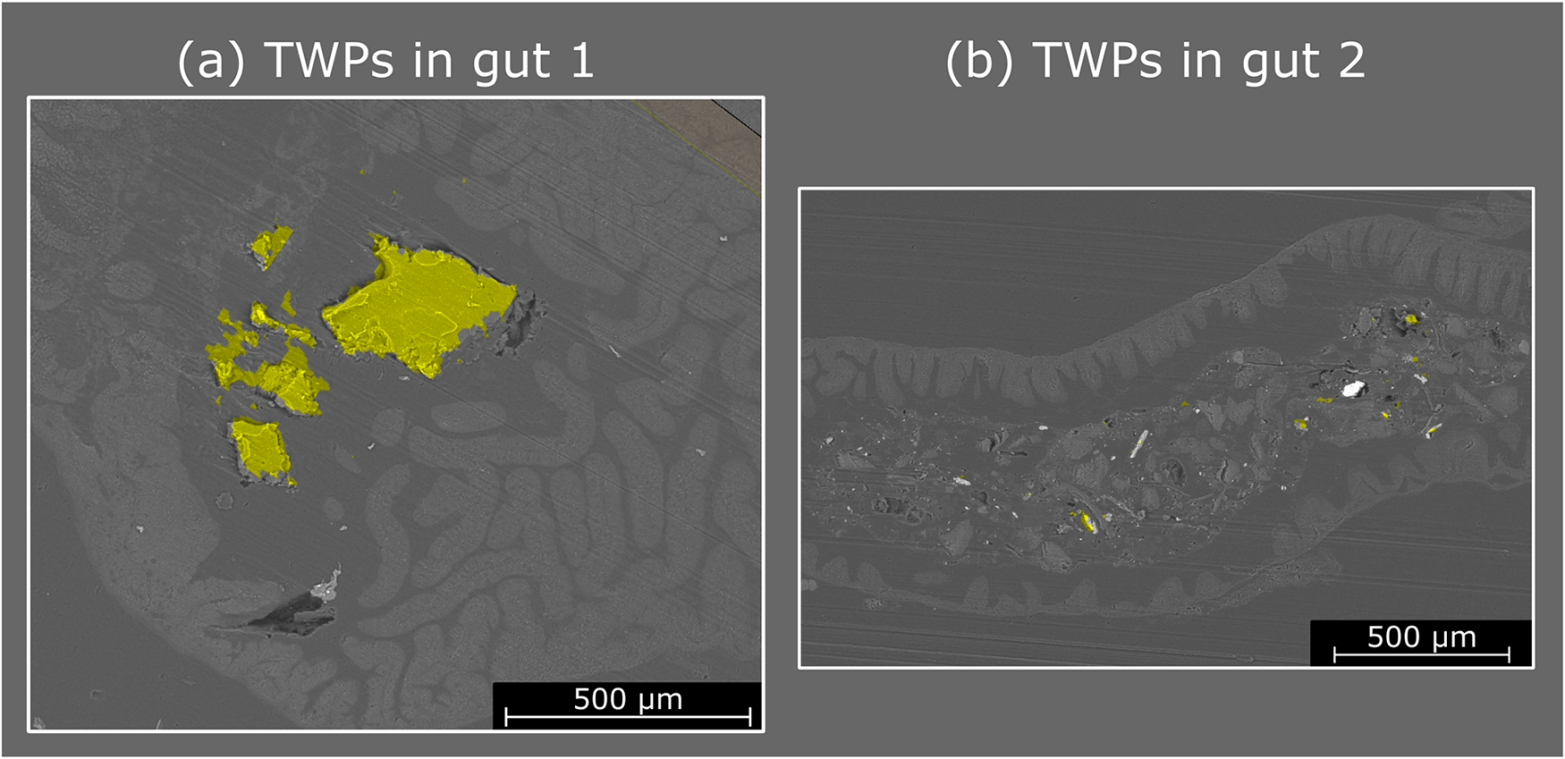
Classification results of images obtained from two different zebrafish guts exposed to TWPs overlaid with SEM pictures (a, b). Pixels classified as TWPS are marked in yellow. Pixels classified as tissue or paraffin are transparent. The area analyzed by LA-ICP-MS was larger than the SEM picture; therefore, the area is not marked.

## Conclusion

Despite their widespread occurrence, the detection of TWPs remains challenging due to their complex and heterogeneous composition, which differs fundamentally from widely studied thermoplastics and thermoset microplastics. Here, we demonstrate that LA-ICP-MS elemental imaging combined with random forest classification provides a powerful approach for detecting TWPs in biotic tissue. While individual marker elements such as Zn or Ti were insufficient to identify TWPs due to overlapping with signals from biological tissue, the multivariate classification model successfully achieved reliable discrimination based on combining multiple marker elements. The developed model showed excellent performance on independent test data, minimizing false positives and accurately identifying TWPs even in the presence of natural particles. Application to zebrafish gut samples confirmed that TWPs could be detected. These findings highlight the potential of elemental fingerprinting combined with machine learning to overcome the limitations of existing analytical methods for TWP monitoring. Beyond advancing microplastic research, this approach provides a framework for studying particle uptake, translocation, and distribution in organisms and humans, contributing to a better understanding of the ecological and health implications of TWPs. The growing regulatory and environmental focus on tire wear emissions underscores the urgent need for advanced analytical methods capable of identifying TWPs with high accuracy in complex matrices. Future instrumental improvements resulting in increased sensitivity may further boost the lateral resolution of the developed method. ICP-TOF-MS-based detection, enabling simultaneous detection of a wide *m*/*z* range, could further improve the classification approach’s robustness and extend the approach to other biological matrices and particles.

## Supplementary Material



## Notes

Results of TWPs size analysis, results of leaching experiments with Xylene, and all raw elemental maps recorded for this study. (PDF)
