# Supplementary material for: A Method for the Detection of Tire Wear Microplastics in Zebrafish Guts by Laterally Resolved LA-ICP-MS-Based Elemental Fingerprinting and Chemometrics
Source: Anal Chem. 2026 Apr 15;98(16):11760–8. doi: 10.1021/acs.analchem.5c07035 (PMC13130160; doi:10.1021/acs.analchem.5c07035)
Supplement: Supplementary file 1 [file ac5c07035_si_001.pdf]

Supporting Information

A method for the detection of tire wear microplastics in zebrafish guts by laterally resolved LA-ICP-MS-based elemental fingerprinting and chemometrics

Lukas Brunnbauer[1,*], Šimon Juračka[1,2], Michaela Vykypělová[3], Lucie Vrlíková[4], Elisabeth Eitenberger[1], Pavel Pořízka[2,5], Ondrej Adamovsky[3], Jozef Kaiser[2,5], Gabriela Kalčíková[6], Andreas Limbeck[1]

[1]TU Wien, Institute of Chemical Technologies and Analytics, Getreidemarkt 9/164-I^{2}AC, Vienna, 1060, Austria

[2]Faculty of Mechanical Engineering (FME), Brno University of Technology, Technická 2896/2, 616 69, Brno, Czech Republic

[3]RECETOX, Faculty of Science, Masaryk University, Kotlářská 2, Brno 611 37, Czech Republic

[4]Institute of Animal Physiology and Genetics, Czech Academy of Sciences, Veveří 97, 602 00 Brno, Czech Republic

[5]Central European Institute of Technology (CEITEC), Brno University of Technology, Purkyňova 656/123, 61200, Brno, Czech Republic

[6]Faculty of Chemistry and Chemical Technology, University of Ljubljana, Večna pot 113, Ljubljana 1000, Slovenia

*corresponding author: Lukas.brunnbauer@tuwien.ac.at

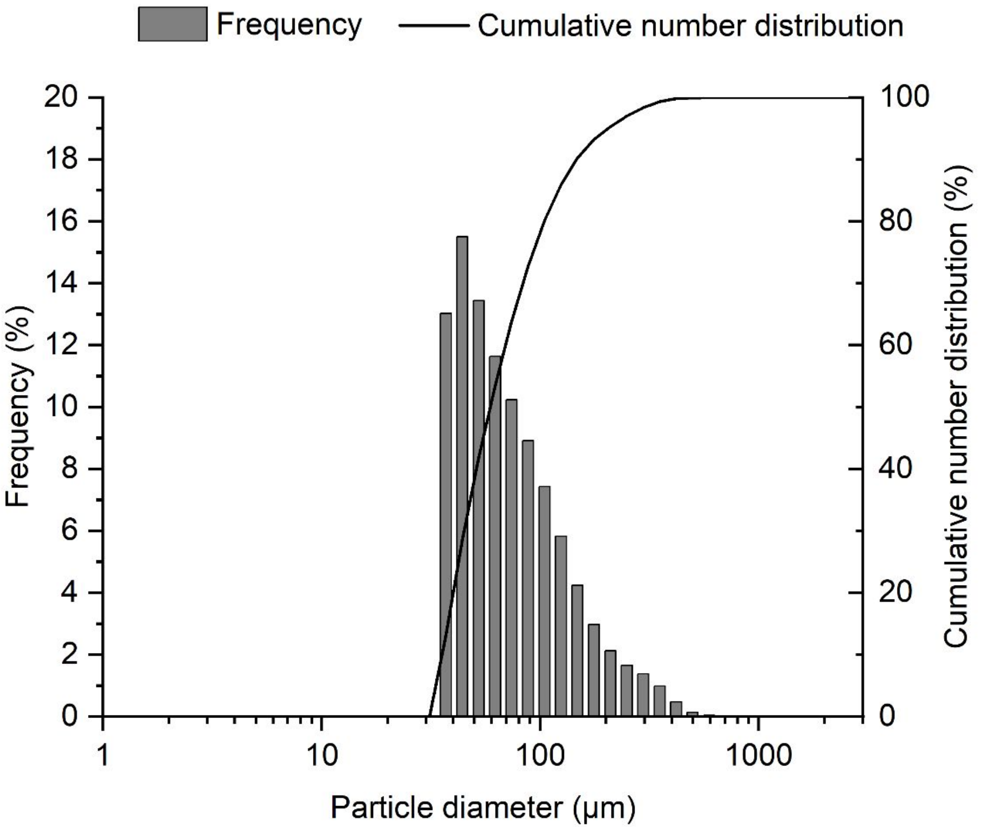

Figure S1: Size distribution of TWPs

Xylene leaching

We leached 100 mg of TWPs in 10 ml of Xylene for 3 hours (the same time the zebrafish are exposed to Xylene during sample preparation) with constant magnetic stirring. Subsequently, the TWPs were separated from the Xylene via centrifugation and dried at 90°C. Next, pristine and leached particles were fixed in paraffin at the same mass fraction and analyzed using LA-ICP-MS with 10 repeat measurements using a spot size of 70 µm, 2.4 J/cm2 fluence, 50 Hz and a scan speed of 700 µm/s. Obtained signals were averaged and plotted with the standard deviation as error bars (Figure S2). It can be seen that exposure to Xylene does not change the elemental composition of the elements under investigation.

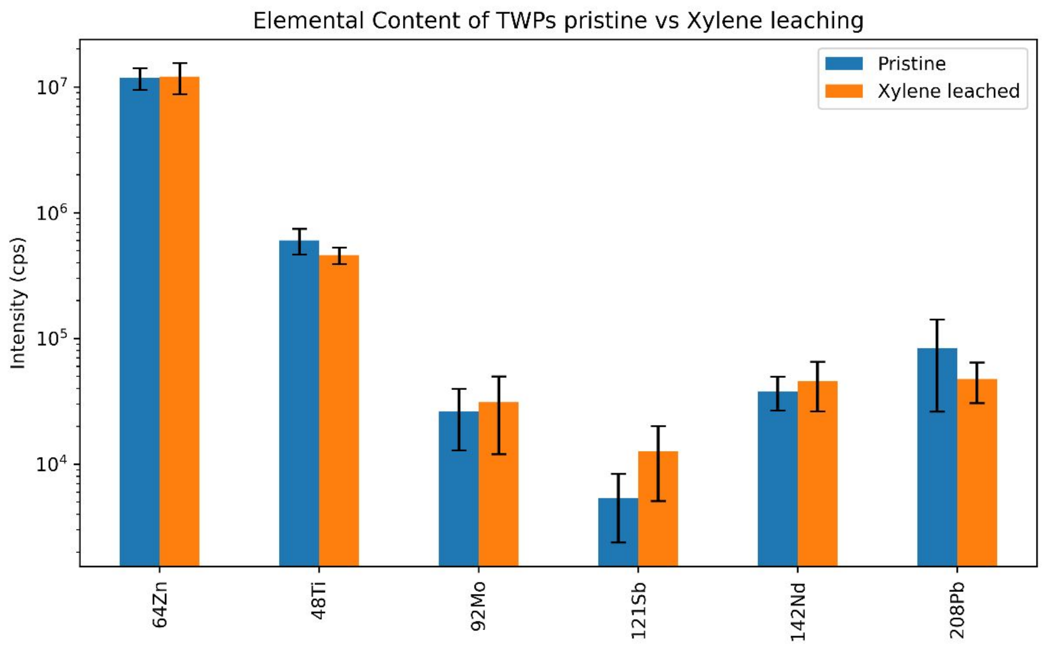

Figure S2: Comparing the elemental composition of pristine TWPs and TWPS leached in Xylene for 3 hours

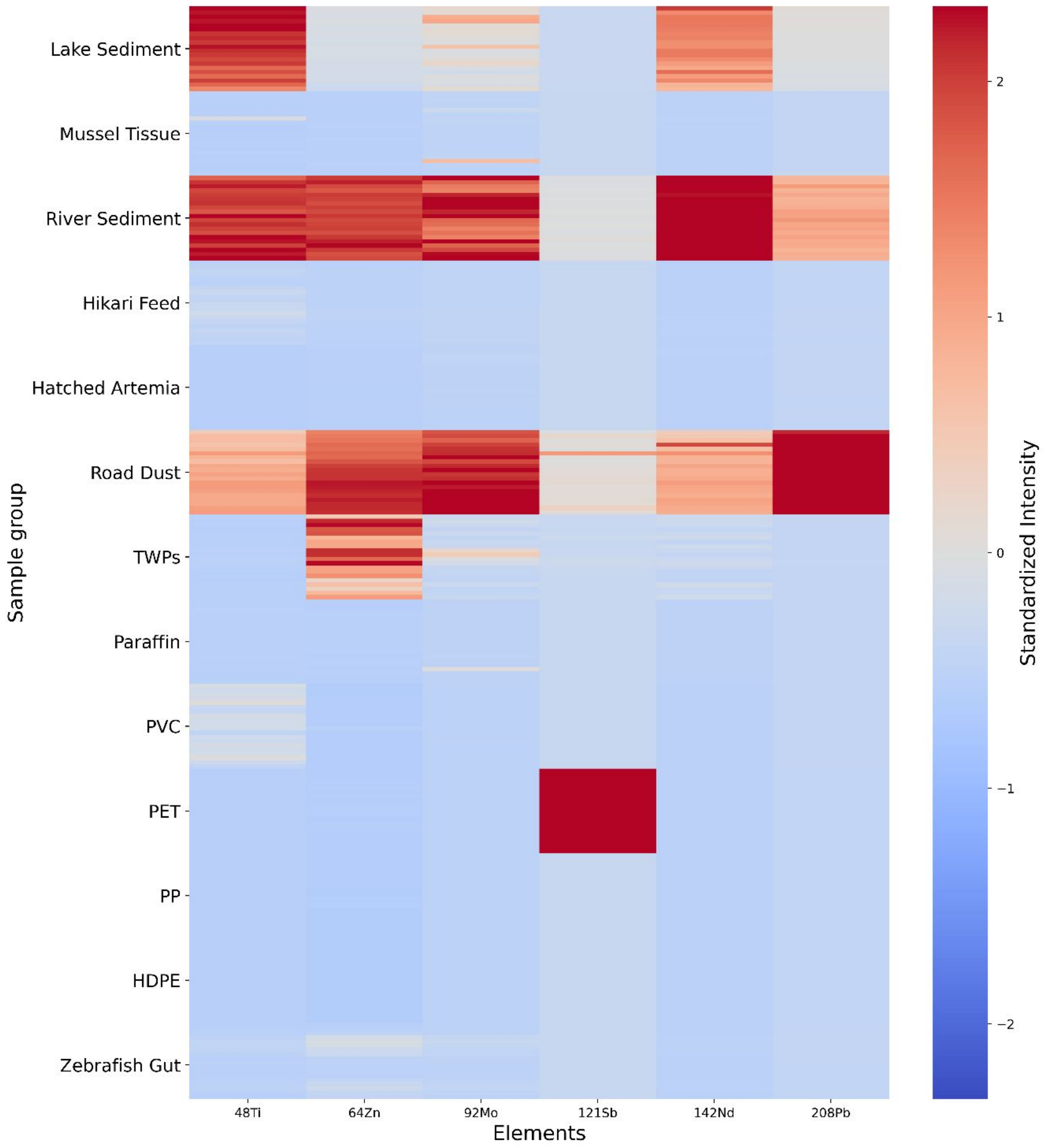

Figure S3: Heatmap showing the elemental patterns for the bulk elemental fingerprint

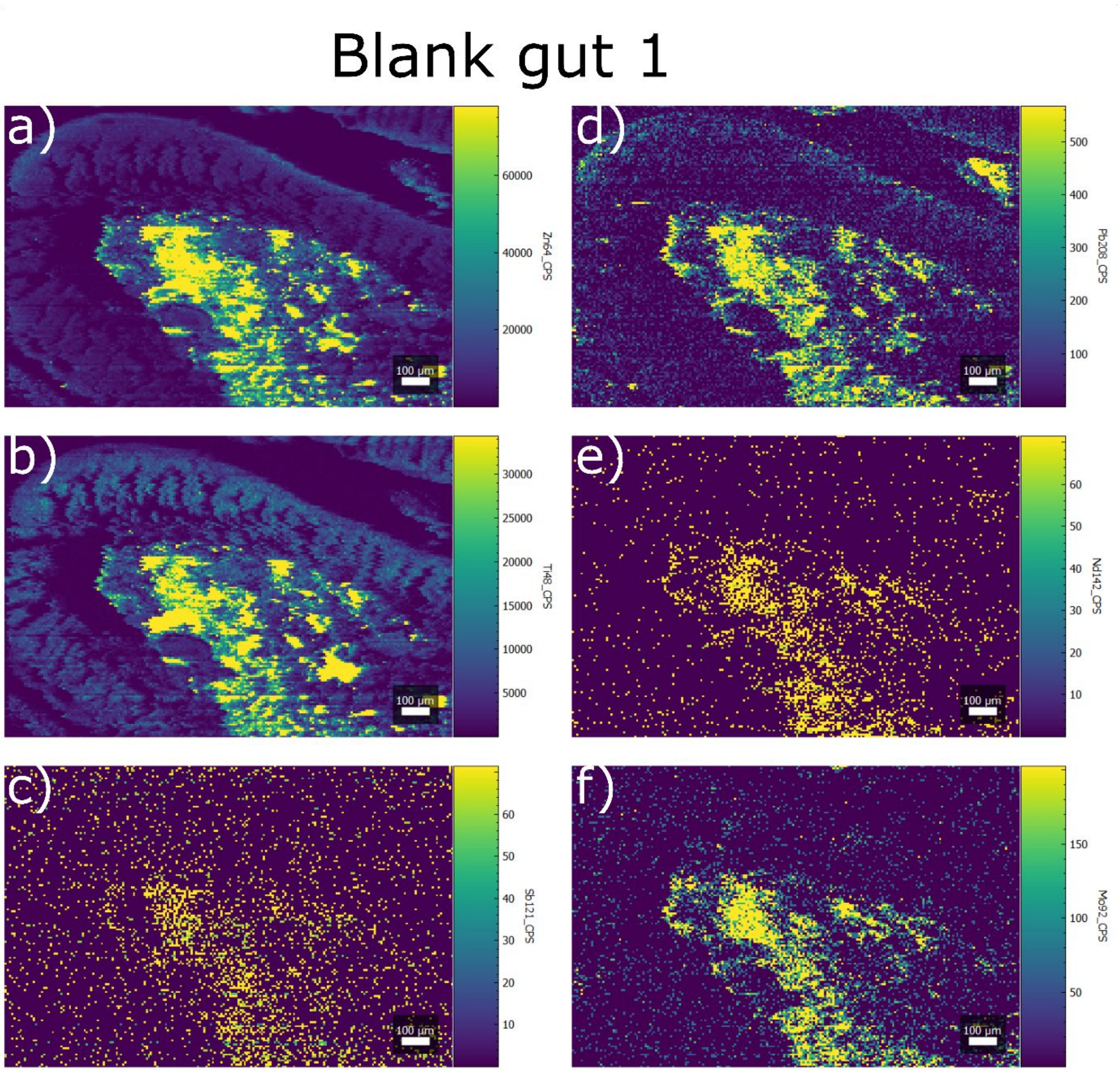

Figure S4: Elemental maps for (a) Zn, (b) Ti, (c) Sb, (d) Pb, (e) Nd, and (f) Mo for the sample Blank gut 1

TWPs 1

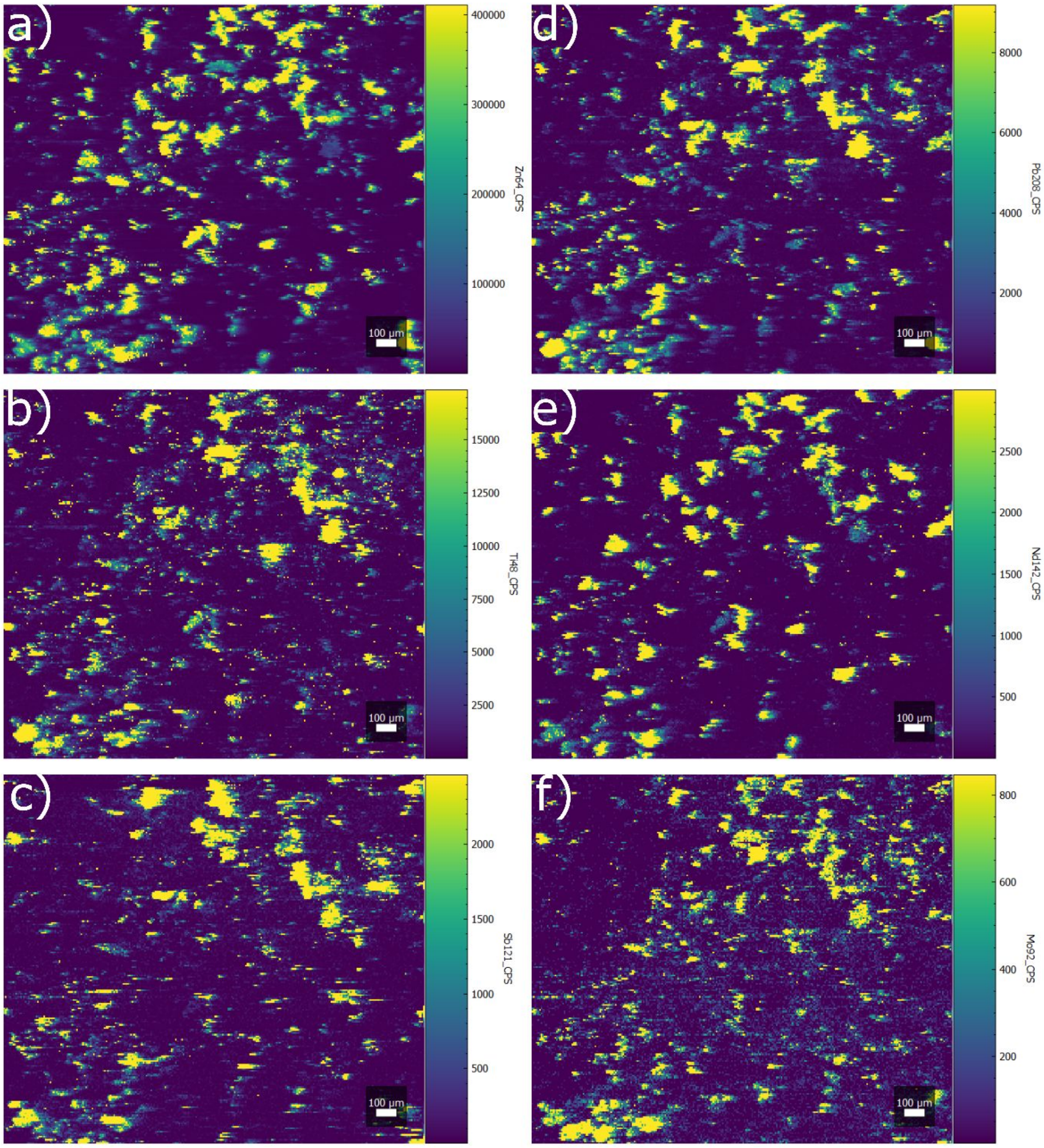

Figure S5: Elemental maps for (a) Zn, (b) Ti, (c) Sb, (d) Pb, (e) Nd, and (f) Mo for the sample TWRP 1

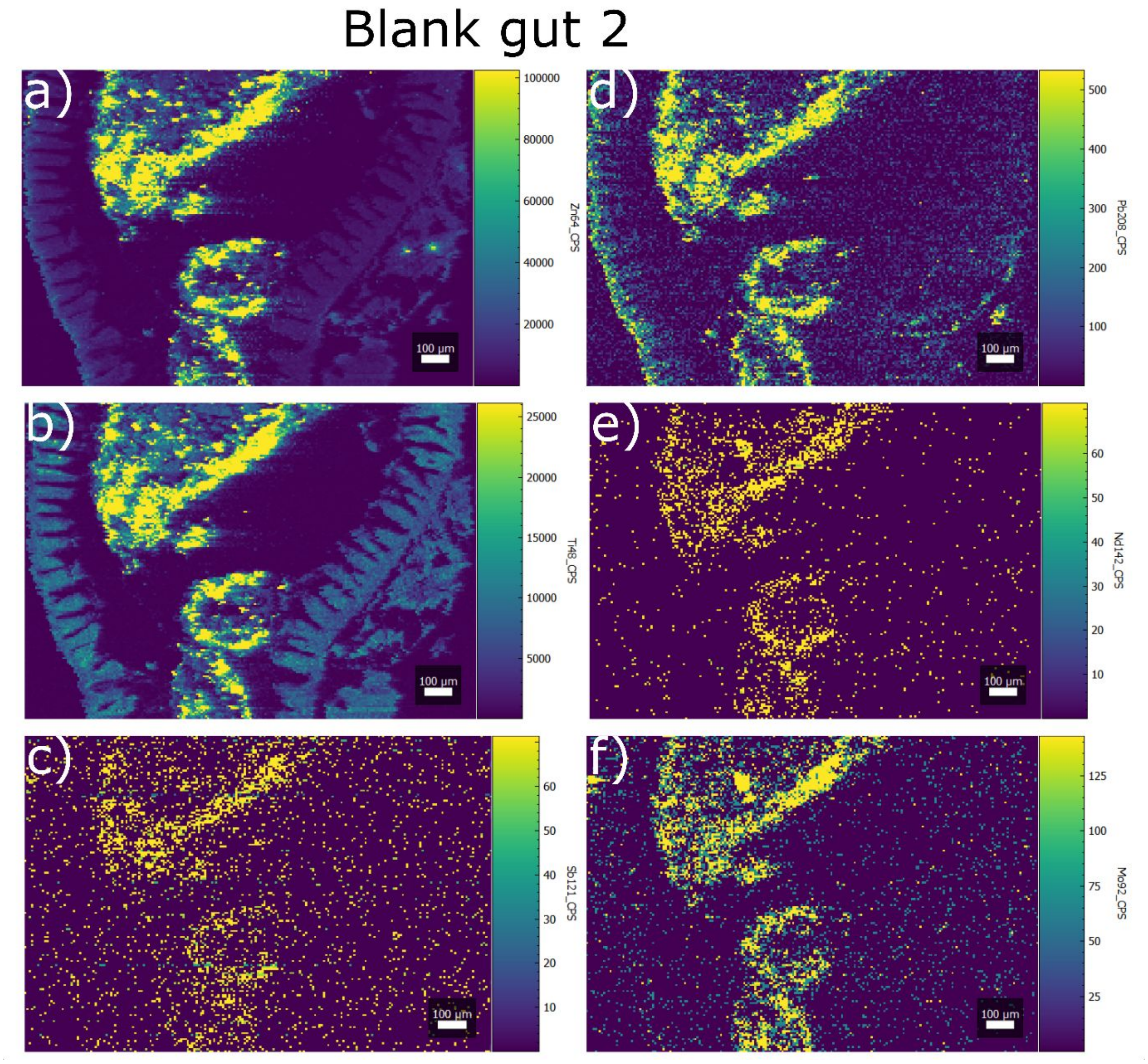

Figure S6: Elemental maps for (a) Zn, (b) Ti, (c) Sb, (d) Pb, (e) Nd, and (f) Mo for the sample Blank gut 2

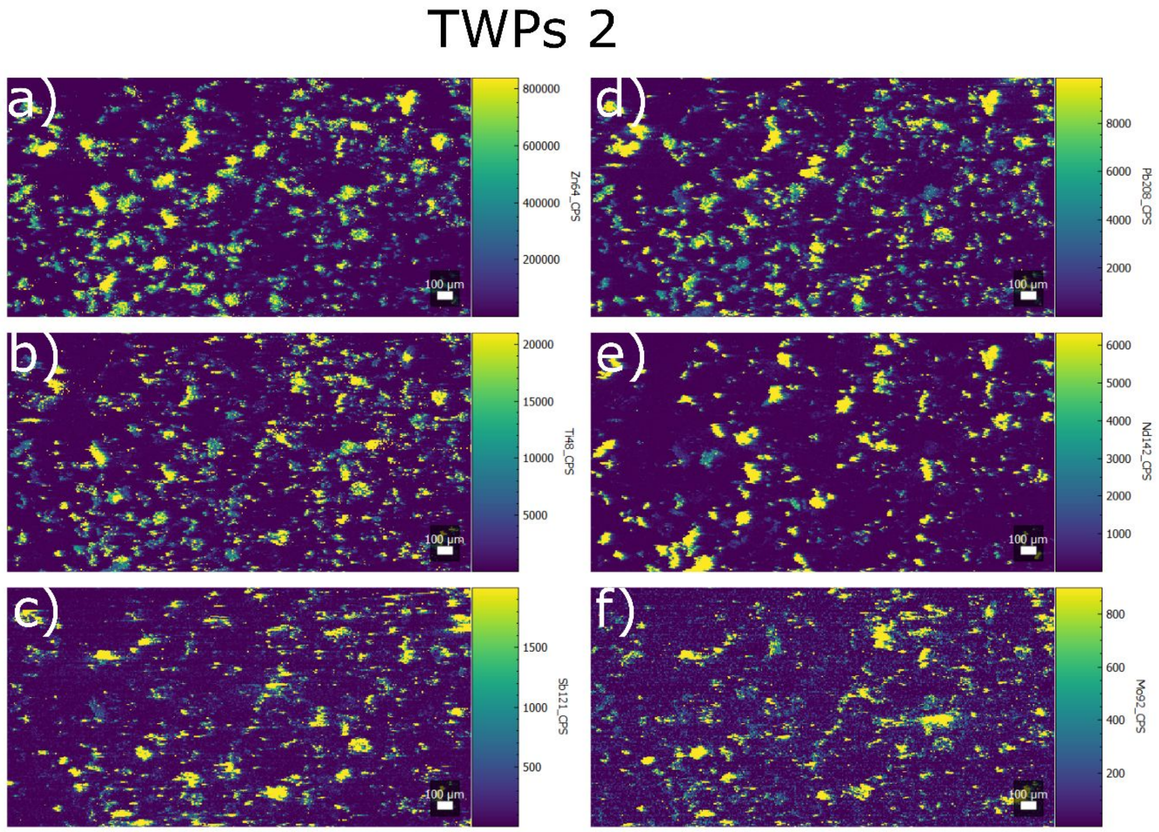

Figure S7: Elemental maps for (a) Zn, (b) Ti, (c) Sb, (d) Pb, (e) Nd, and (f) Mo for the sample TWRP 2

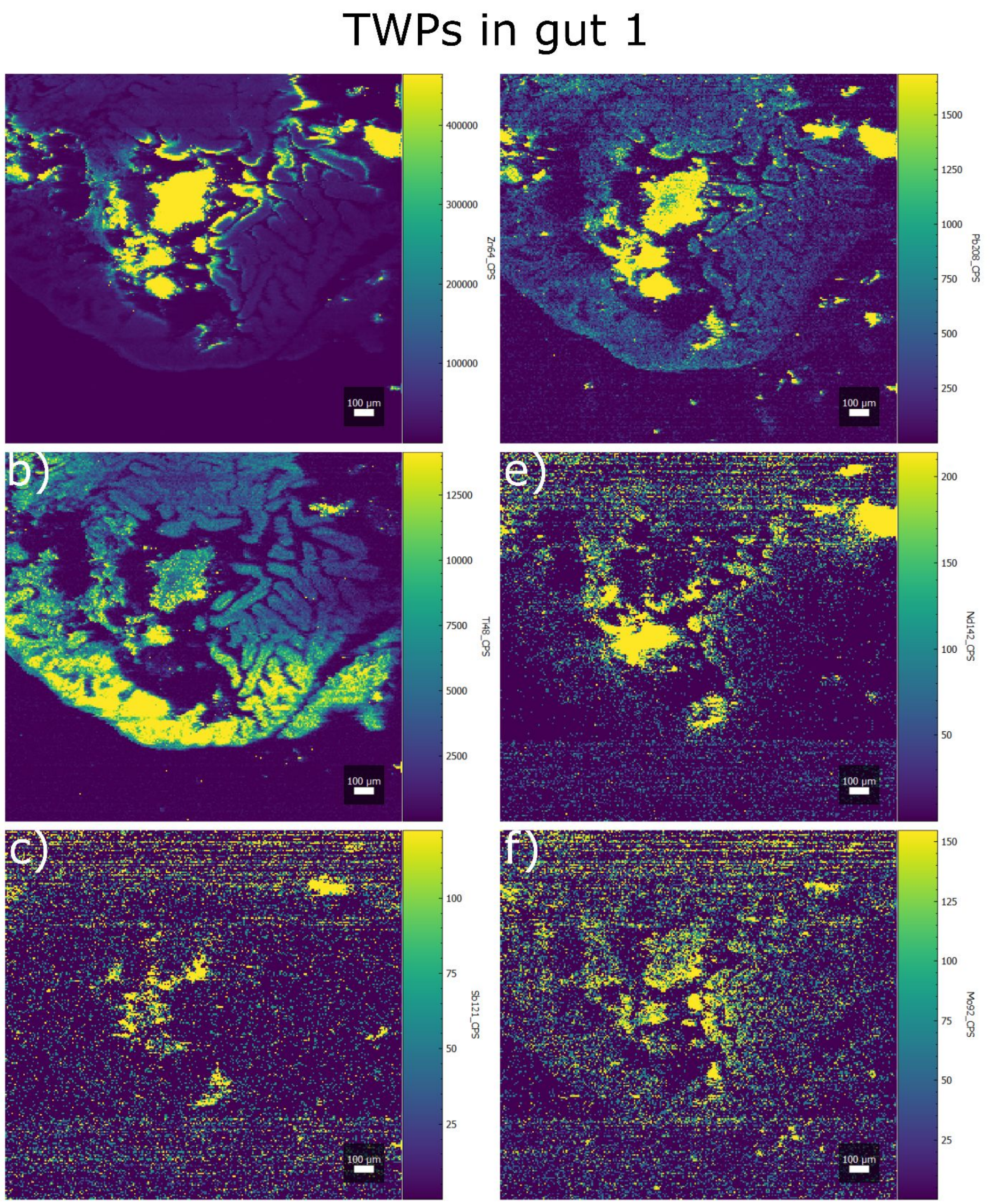

Figure S8: Elemental maps for (a) Zn, (b) Ti, (c) Sb, (d) Pb, (e) Nd, and (f) Mo for the sample TWRP in gut 1

TWPs in gut 2

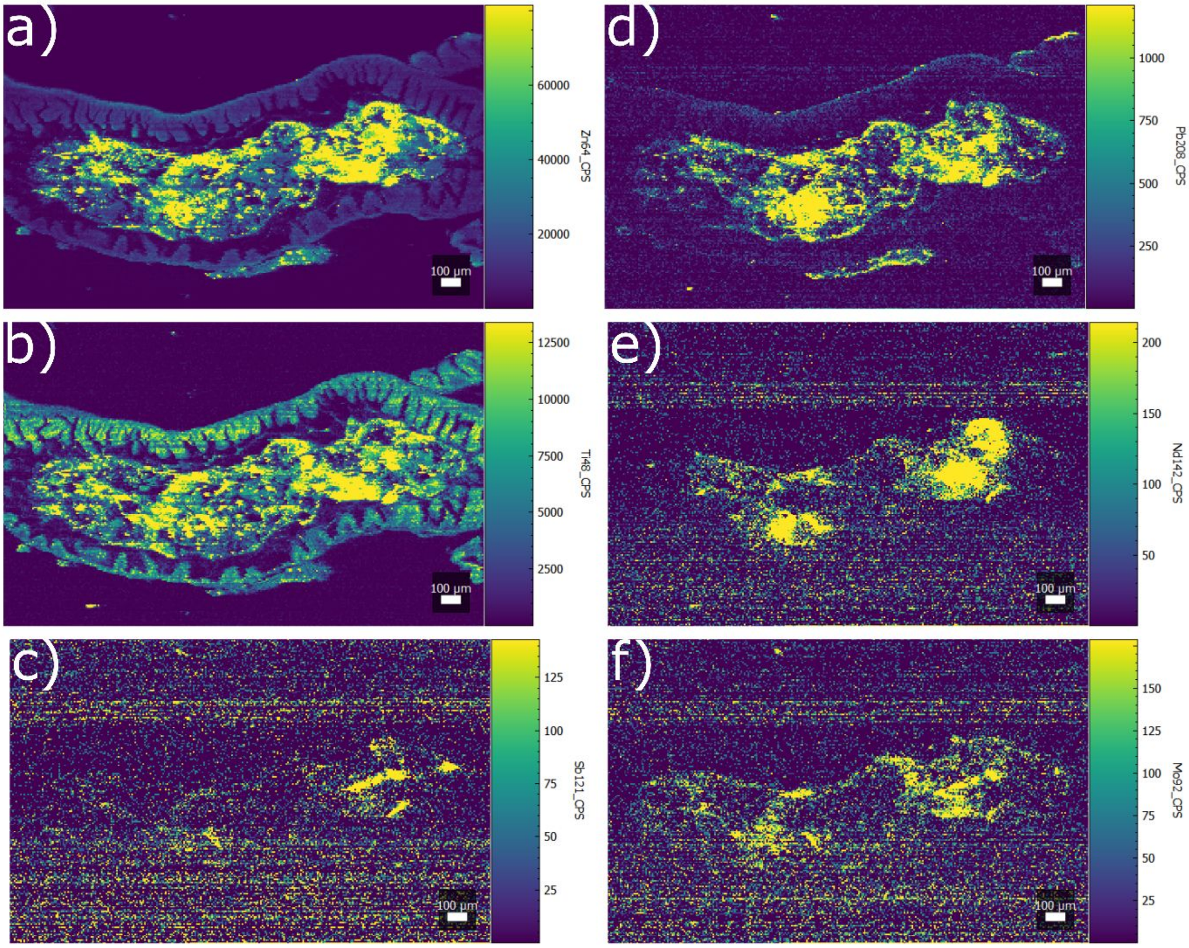

Figure S9: Elemental maps for (a) Zn, (b) Ti, (c) Sb, (d) Pb, (e) Nd, and (f) Mo for the sample TWRP in gut 2
